# Psychosocial distress in rural palliative care: Preliminary longitudinal findings using the DADDS

**DOI:** 10.1017/S1478951525100813

**Published:** 2025-10-29

**Authors:** Geena Bennett, Felicity Bates, Kerith Duncanson, Ian Heslop, Jennifer Schneider, Sarah Dineen-Griffin

**Affiliations:** 1 School of Medicine and Public Health, College of Health, Medicine and Wellbeing, University of Newcastle, Newcastle, NSW, Australia; 2 Coffs Clinical Network Pharmacy Department, Senior Clinical Pharmacist, Mid North Coast Local Health District, Coffs Harbour, NSW, Australia; 3 School of Biomedical Sciences and Pharmacy, College of Health, Medicine and Wellbeing, University of Newcastle, Newcastle, NSW, Australia; 4 Clinical Pharmacology and Clinical Toxicology, School of Medicine and Public Health, College of Health, Medicine and Wellbeing, University of Newcastle, Newcastle, NSW, Australia

**Keywords:** Palliative care, psychosocial distress, rural health, death and dying distress scale, survival analysis

## Abstract

**Objectives:**

Palliative care enhances life, but rural Australia faces significant inequities, and psychosocial distress, an important yet often overlooked aspect, is under-recognized in these settings. This study examines how psychosocial distress evolves in rural palliative patients using the Death and Dying Distress Scale (DADDS).

**Methods:**

A longitudinal study was conducted with palliative care patients in rural hospitals on Australia’s east coast. Distress levels were measured using DADDS at multiple timepoints. Mixed-effects models assessed distress trajectories, while survival analyses (Weibull model) examined whether average distress changes predicted survival duration. For comparability, DADDS scores in mixed-effects models were standardized (0–100%), whereas survival analyses used raw total score changes.

**Results:**

Adjusted mean total DADDS was 37.14 ± 22.67, with highest distress in *fear of suffering and pain* (49.95 ± 26.56) and lowest in *fear of sudden death* (30.26 ± 30.24). Distress followed a U-shaped trajectory: peaking early (52.68), declining mid (29.85) and late stages (28.26), then rising near death (53.05) (EMMs). Statistically significant changes included declines from early to mid-stage (β = −22.84, p = 0.007) and increases from late to near-death (β = 24.79, p = 0.003). Distress increased most from late to near-death in fear of suffering and death (β = 27.38, p = 0.006) and declined most from early to mid-stage in fear of dying (β = 28.01, p = 0.007). Higher distress correlated with shorter survival; each one-point increase in distress linked to a 6.97% survival reduction (time ratio = 0.930, β = −0.070, p < 0.001).

**Significance of results:**

Psychosocial distress peaks in early palliative care and near death and is associated with reduced survival. Support should prioritize fears of suffering and pain during these stages, address fear of the dying process earlier, and remain attentive to persistent concerns such as loss of time and opportunity.

## Introduction

Palliative care is a human right that aims to improve the quality of life for individuals with life-limiting illnesses by addressing their physical, emotional, psychological, social, and spiritual needs (AIHW 2024). Prioritizing comfort over curative treatments, it seeks to relieve suffering and enhance well-being for patients, families, and caregivers. However, its access and service delivery remain highly inequitable outside of metropolitan centers, particularly in addressing psychosocial needs (Parliament of NSW 2022).

In Australia, the demand for palliative care is rising due to an ageing population and increasing prevalence of cancer and other chronic illnesses (Parliament of New South Wales 2022). While approximately 30% of Australians reside outside metropolitan centers, only 16% of palliative care specialists practice in these areas. Rural and remote regions face ongoing challenges such as workforce shortages and limited resources, which contribute to fragmented services. Limited mental health support further exacerbates psychological distress for patients and caregivers, compounding existing inequities (Parliament of New South Wales 2022).

Historically, palliative care research has prioritized management of physical symptoms, with less emphasis on the psychosocial distress, particularly in rural populations (Boston et al. 2011; Sultana et al. 2021). Patients frequently experience high levels of psychosocial distress, especially existential, yet research suggests that this distress is significantly underdiagnosed, undertreated and poorly understood (Thekkumpurath et al. 2008). Moreover, despite evidence suggesting that these psychosocial symptoms can be as debilitating as physical ones, they remain under-recognized in clinical practice, particularly in rural settings with limited mental health resources (Boston et al. 2011; Parliament of NSW 2022).

Prior research has indicated links between distress and poorer patient outcomes in advanced illness (Kiecolt-Glaser and Glaser 1999; Khalid et al. 2023). Furthermore, emerging evidence suggests that addressing psychosocial distress through targeted interventions can significantly improve patient well-being. Psychological interventions aimed at existential distress in terminally ill patients have shown potential benefits in enhancing quality of life and preventing further deterioration in mental health (Seiler et al. 2024). Specifically, Lo et al. reported that individualized psychotherapy interventions reduced depressive symptoms and increased spiritual well-being in advanced cancer patients (Lo et al. 2014). Such interventions may alleviate physical symptoms like pain, fatigue, dyspnea, and insomnia (Von Blanckenburg and Leppin 2018), with meta-analysis even suggesting some potential survival benefits (Smith et al. 2021). Beyond individual well-being, distress is linked to unmet palliative needs and increased healthcare utilization (Hildenbrand et al. 2022). Rural investment in psychosocial palliative care can reduce this burden, strengthen the workforce, and improve outcomes through targeted clinician training (Ghoshal 2025).

Currently, there is no consensus on the clinical threshold that defines pathological existential distress, and many rural hospitals in Australia lack standardized tools to assess it (Vehling and Kissane 2018). The Palliative Care Outcomes Collaboration (PCOC) Palliative Care Problem Severity Scale (PCPSS) is a commonly used clinician-rated tool to record psychological/spiritual distress. However, the tool focuses primarily on physical symptom burden, lacks dedicated metrics for psychosocial distress, and contains no guidelines on how to rate distress. Another tool recently implemented at some sites is the Psycho-Existential Symptom Assessment Scale (PeSAS), but its uptake has been hindered by extensive staff training requirements resource constraints from training barriers (Kissane et al. 2022). In contrast, the Death and Dying Distress Scale (DADDS) is a validated instrument that can be self-completed by patients or administered through interviews by palliative care workers, offering a more accessible approach to measuring existential distress (Lo et al. 2011).

The DADDS specifically assesses death anxiety and related psychosocial concerns in palliative patients. It is a 15-item scale summarized into two core domains: psychosocial fears about death and fears of the dying process (Shapiro et al. 2021; Walbaum et al. 2024). Further categorization has identified subscales assessing fear of lost time and opportunity, an uncertain future, suffering and pain, and sudden death (Vehling et al. 2017; An et al. 2018). Research using the DADDS has demonstrated its high reliability, with validation studies showing that 45% of patients score within the higher distress range (Lo et al. 2011). Additionally, research utilizing the DADDS suggests that death anxiety, is more prevalent among younger patients, females, and those earlier in their diagnosis (Walbaum et al. 2024). However, little is known about how distress measured by the DADDS evolves over time in palliative care populations, particularly in rural settings.

This study aims to examine how distress levels evolve over time among rural palliative care patients, using the DADDS to track psychosocial trajectories throughout care. By identifying critical periods when distress peaks, this research seeks to inform the timing and nature of psychosocial interventions in rural palliative care settings. We hypothesize that distress levels are highest early in the palliative timeline, when patients are first coming to terms with their diagnosis, and gradually decline as patients adjust and receive support. Ultimately, the aim is that by improving the recognition and management of psychosocial distress in rural palliative care, this may enhance quality of life and contribute to more equitable healthcare outcomes.

## Methods

### Design

A longitudinal study was conducted as a sub-study of a larger longitudinal prospective cohort study.

### Setting

The study was conducted from October 2022 to April 2025 in three public hospitals within a rural coastal Australian health district, encompassing Local Government Areas classified under the Modified Monash (MM) Model as between MM 3 (regional centers) and MM 7 (extremely remote areas) (Department of Health and Aged Care 2024). In 2021, the district served an estimated population of 226,422 residents, with projections indicating growth to approximately 241,184 by 2031 (NSW Health n.d.). Notably, the district has a higher proportion of residents aged 65 and over (30% compared to the NSW average of 18%), contributing to a significant demand for primary and palliative care services (ABS 2021).

### Participants

Participants were inpatients or outpatients receiving palliative care at participating hospitals between October 2022 and April 2025. Eligibility required being 18 years or older, having a life-limiting illness, an Australian-modified Karnofsky Performance Score (AKPS) score of ≥30, and palliative care input and completed two or more DADDS. Patients were excluded if they lacked the cognitive or language ability to provide consent or complete the DADDS.

### Data collection and measures

Demographic and medical data were obtained from electronic medical records and clinician-administered DADDS questionnaires, completed either on paper or electronically. All data were stored securely in a password-protected REDCap (Research Electronic Data Capture) project hosted by the Hunter Medical Research Institute (HMRI) (Harris et al. 2009, 2019). Data were de-identified prior to export for analysis.

Collected demographics included gender, age, primary diagnosis, known metastases (if applicable), and near-existing pain, anxiety, or depression. Medical data recorded on the first DADDS survey day included AKPS score and PCOC phase (stable, unstable or deteriorating).

The DADDS was used to assess psychosocial distress related to death and dying. It consists of 15 items rated on a 6-point Likert scale from 0 (no distress) to 5 (extreme distress), with a total score ranging from 0 to 75 (Lo et al. 2011) The DADDS was administered by a palliative care worker at study enrolment and subsequently at approximately monthly intervals until the participant’s death, withdrawal, or loss to follow-up. In addition to total scores, its six conceptually derived subscales were analyzed: psychosocial fears about death (10 items; Q1–10), fear of the dying process (5 items; Q11–15), fear of loss of time and opportunity (4 items; Q1–3, 6), fear of an uncertain future (5 items; Q4–5, 7, 9, 10), fear of suffering and pain (3 items; Q8, 12, 14), and fear of sudden death (3 items; Q11, 13, 15) (An et al. 2018).

Standardized DADDS total and subscale scores were calculated by dividing raw scores by the maximum possible score for each respective scale (i.e., 5 multiplied by the number of items and multiplying by 100). This produced scores on a 0–100 scale, representing the percentage of each scale’s theoretical maximum. This approach enabled direct comparison across subscales and facilitated interpretation of changes in distress over time. Baseline scores were defined as the first recorded DADDS total and subscale scores for each participant.

To examine distress across the care trajectory, time was categorized into four stages using a combination of conceptual and data-driven methods. The pre-death category was defined by proximity to death, comprising surveys completed within 30 days of the participant’s death. The remaining three categories, early, mid, and late, were derived using quartile-based distributions and change point detection, then adjusted to align with whole-month intervals to enhance interpretability. Specifically, these were; early stage: within first 3 months of palliative care, mid stage: 3–10 months, late stage: ≥10 months. Time in palliative care was calculated from the date of each participant’s initial PCOC assessment to each subsequent DADDS survey.

### Ethics approval

This study was approved by the [region blinded] New South Wales Human Research Ethics Committee (Approval Number: 2022/ETH00648), with site-specific authorizations granted for data collection at participating institutions (2022/STE01913 [blinded], 2023/STE01119 [blinded]).

### Data analysis

Descriptive statistics were used to summarize demographics, baseline medical characteristics, and DADDS scores. Frequencies and percentages were reported for categorical variables, while means with standard deviations (SD) or medians with interquartile ranges (IQR) were presented for continuous variables, as appropriate. Cross-tabulations were used to calculate frequencies. Both raw and standardized scores for the total DADDS and its subscales were reported, with standardized scores expressed on a 0–100% scale representing the percentage of their maximum possible value to air comparable interpretation.

To assess changes in distress over time, linear mixed-effects models were fitted for total and subscale DADDS scores across the palliative care trajectory. This approach accommodated unbalanced, irregularly timed data and accounted for within-subject correlations from repeated measures. Time category (early, mid, late, near-death) was included as an ordinal fixed effect, with participant ID modelled as random intercepts to capture individual variability. A random slope for time was trailed but omitted due to model complexity relative to sample size affecting fit. A heterogeneous compound symmetry covariance structure was specified, with time as the repeated measure and participant ID as the subject. Estimated marginal means (EMMs) were extracted for each time category to provide model-adjusted distress estimates. Standardized scores, expressed as percentages of their maximum possible values (0–100%), were used consistently for comparability.

As the pre-death period is defined by proximity to death rather than a linear continuation of care, Tukey’s HSD test was used to report post-hoc pairwise comparisons between the near-death and early/mid categories. Although the mixed model’s linear contrasts remain valid, these additional comparisons were reported to illustrate potential non-linear transitions of clinical relevance and focused solely on the DADDS total score to demonstrate overall patterns of change while prioritizing clarity and focus on key findings.

A parametric Weibull survival regression model examined the association between changes in total distress scores over the study period and survival time, defined as days from study entry to death or censoring (censoring applied for participants alive or lost to follow-up at study end). Both model-derived expected survival time and observed mean survival time were used to interpret effect sizes. Raw DADDS score changes (scale 0–75) were used and reported for the survival model.

Both the mixed-effects and survival models were adjusted for key covariates: prior elapsed duration in palliative care, survey interval duration, relative distress at study entry (initial DADDS score), age, gender, primary diagnosis, and PCOC phase. Continuous covariates were mean-centered and scaled; prior palliative care duration and survey interval were log-transformed to reduce skewness.

Model assumptions for both the linear mixed-effects and survival models were checked and satisfied. Model fit was evaluated using Akaike Information Criterion (AIC) and visual diagnostic plots. All analyses were conducted using R (version 4.4.2) and JMP (version 17) (R Core Team 2023; SAS Institute Inc 2023). Sample size and power calculations were performed using G*Power (version 3.1.9.7) (Faul et al. 2020). Significance was set at α = 0.05 with power β = 0.80.

## Results

### Demographic characteristics of study sample

There were 20 participants. The mean (SD) age was 68.35 (SD 9.28) years, and 55% were female. Cancer was the primary diagnosis in 75%, with 86.7% of those people also having known metastatic disease at recruitment. The remaining 25% had end-stage organ disease. Median AKPS at baseline was 45 (IQR 40–60), with 75% in a stable PCOC phase and 25% deteriorating. A summary of the demographic characteristics is provided in Table 1.

**Table 1.** Sample demographics (N = 20)

		n (%)
Patient characteristics	Gender	
	Male	9 (45)
	Female	11 (55)
	Age	
	Mean ± SD	68.35 ± 9.28
	DADDS completed	
	Median (IQR)	2 (2–4)
Diagnosis characteristics	Primary diagnosis	
	Cancer	15 (75)
	Known metastases	13 (86.7)
	End-stage disease	5 (25)
	Cancer; primary-type	
	Blood	1 (6.7)
	Breast	4 (26.7)
	Endocrine	1 (6.7)
	Gastrointestinal	1 (6.7)
	Genitourinary	3 (20)
	Gynecological	1 (6.7)
	Lung/Respiratory	2 (13.3)
	Other/Unknown primary	2 (13.3)
	End-stage disease; type	
	Heart	2 (40)
	Liver	1 (20)
	Pulmonary	2 (40)
	Palliative care duration until death^	
	Median (IQR), days	205 (157–414.5)
Baseline characteristics	Co-morbidities	
	Pain	1 (5)
	Anxiety	2 (10)
	Depression	2 (10)
	AKPS score	
	40	10 (50)
	50	2 (10)
	60	8 (40)
	Phase	
	Stable	17 (85)
	Deteriorating	3 (15)

Abbreviation: DADDS, Death and Dying Distress Scale.

Reported as n (%) unless stated otherwise.

^ If deceased (n = 17).

### DADDS characteristics

Sixty-three DADDS surveys were completed across four palliative care timepoints. Total scores (observed, unadjusted) ranged from 0 to 64, with a mean (SD) of 27.86 (17.00), indicating substantial inter-individual variability in death-related distress. The lowest mean total score of 23.79 (SD) occurred during the mid-stage, with the highest being 31.74 (SD) during the late stage. When standardized as a percentage of maximum possible score per subscale, the highest overall distress was reported in *fear of suffering and pain* (49.95%), and the lowest in *fear of sudden death* (30.26%). Descriptive statistics for total and subscale scores at each timepoint are presented in Table 2.

**Table 2.** DADDS characteristics

	Early (n = 15) M ± SD		Mid (n = 19) M ± SD		Late (n = 19) M ± SD		^Near-death (n = 10) M ± SD		Overall (n = 60) M ± SD	
	Raw	Adjusted	Raw	Adjusted	Raw	Adjusted	Raw	Adjusted	Raw	Adjusted
Total score	25.67 ± 21.67	34.22 ± 28.89	23.79 ± 14.67	31.72 ± 19.56	31.74 ± 14.20	42.32 ± 18.93	31.50 ± 18.39	42.00 ± 24.52	27.86 ± 17.00	37.14 ± 22.67
Subscale 1: Psychosocial fears about death	16.53 ± 15.15	33.07 ± 30.29	15.74 ± 9.51	31.47 ± 19.02	22.74 ± 8.87	45.47 ± 17.74	21.50 ± 10.96	43.00 ± 21.91	18.95 ± 11.33	37.90 ± 22.66
Subscale 2: Fear of dying process	9.13 ± 7.14	36.53 ± 28.56	8.05 ± 6.60	32.21 ± 26.42	9.00 ± 6.36	36.00 ± 25.44	10.00 ± 7.75	40.00 ± 30.98	8.90 ± 6.71	35.62 ± 26.85
Subscale 3: Fear of loss of time & opportunity	5.87 ± 6.45	29.33 ± 32.23	5.95 ± 3.75	29.74 ± 18.74	7.89 ± 3.57	39.47 ± 17.86	6.80 ± 4.57	34.00 ± 22.83	6.65 ± 4.58	33.25 ± 22.90
Subscale 4: Fear of an uncertain future	8.133 ± 7.61	32.53 ± 30.46	7.05 ± 5.03	28.21 ± 20.12	10.95 ± 5.10	43.79 ± 20.42	11.40 ± 6.45	45.60 ± 25.80	9.17 ± 6.12	36.70 ± 24.47
Subscale 5: Fear of suffering & pain	6.93 ± 4.08	46.22 ± 27.19	7.26 ± 4.62	48.42 ± 30.78	8.11 ± 3.68	54.04 ± 24.54	7.60 ± 3.50	50.67 ± 23.35	7.49 ± 3.98	49.95 ± 26.56
Subscale 6: Fear of sudden death	4.73 ± 5.08	31.56 ± 33.85	3.53 ± 3.72	23.51 ± 24.78	4.79 ± 4.21	31.93 ± 28.07	5.70 ± 5.89	38.00 ± 39.26	4.54 ± 4.54	30.26 ± 30.24

Abbreviation: DADDS, Death and Dying Distress Scale.

Reported as percentage of possible maximum score per total or scale.

^ Within <30 days of deceased date.

### Trends in DADDS over time

Linear mixed-effects modelling revealed a statistically significant effect of time on total DADDS scores after adjusting for age, gender, diagnosis, phase of care, baseline distress, duration in palliative care, and interval between surveys (F = 4.57, p = 0.014; Tables 3 and 4). Model-estimated marginal means (as a percentage of maximum possible score) showed a U-shaped pattern: scores were highest during the early stage (52.68%), declined in the mid (29.85%) and late (28.26%) stages, and increased again in the final 30 days before death (53.05%). Statistically significant changes occurred from early to mid-stages (β = −22.84, p = 0.007) and from late to near-death (β = 24.79, p = 0.003); no statistically significant difference was observed between mid and late stages. As expected, higher baseline distress and shorter time in care at baseline were significantly associated with higher scores across timepoints (p < 0.05). Other covariates were not significantly associated with DADDS scores.

**Table 3.** DADDS trends over time periods

	Overall time effect	Mid vs early	Late vs mid	^Pre-death vs late
Timepoint	F	β (SE)	β (SE)	β (SE)
Total score	F = 4.57	−22.84 (7.99)	−1.59 (6.71)	24.79 (7.79)
	p = 0.014*	p = 0.007**	p = 0.815	p = 0.003**
Subscale 1: Psychosocial fears about death	F = 4.24	−21.37 (8.17)	−0.91 (6.75)	24.85 (7.95)
	p = 0.013*	p = 0.013*	p = 0.894	p = 0.004**
Subscale 2: Fear of dying process	F = 3.68	−28.01 (9.79)	−3.01 (8.25)	26.26 (10.15)
	p = 0.023*	p = 0.007**	p = 0.718	p = 0.016*
Subscale 3: Fear of loss of time & opportunity	F = 3.41	−12.02 (11.54)	−3.28 (9.84)	19.79 (8.40)
	p = 0.024*	p = 0.302	p = 0.741	p = 0.023*
Subscale 4: Fear of an uncertain future	F = 3.79	−27.83 (9.87)	1.58 (7.49)	27.07 (10.22)
	p = 0.020*	p = 0.008**	p = 0.834	p = 0.014*
Subscale 5: Fear of suffering & pain	F = 4.20	−24.01 (8.73)	−1.27 (7.69)	27.38 (9.16)
	p = 0.014*	p = 0.009**	p = 0.869	p = 0.006**
Subscale 6: Fear of sudden death	F = 3.10	−26.19 (10.13)	−4.37 (8.11)	24.66 (10.39)
	p = 0.043*	p = 0.014*	p = 0.593	p = 0.028*

Abbreviation: DADDS, Death and Dying Distress Scale.

Reported as percentage of possible maximum score per total or scale.

^ Within <30 days of deceased date.

Significance

* p < 0.05, **p < 0.01.

**Table 4.** Model-derived estimated marginal means of DADDS scores

	Early (M ± SE; 95% CI)	Mid (M ± SE; 95% CI)	Late (M ± SE; 95% CI)	^Near-death (M ± SE; 95% CI)
Total score	52.68 ± 5.61	29.85 ± 5.71	28.26 ± 6.70	53.05 ± 5.52
	[40.95, 64.42]	[18.11, 64.42]	[14.76, 41.76]	[41.09, 65.01]
Subscale 1: Psychosocial fears about death	51.07 ± 5.67	29.70 ± 5.73	28.79 ± 6.65	53.64 ± 5.64
	[39.12, 63.01]	[17.90, 41.50]	[15.39, 42.19]	[41.29, 66.00]
Subscale 2: Fear of dying process	57.30 ± 6.68	29.29 ± 7.04	26.28 ± 8.40	52.54 ± 7.62
	[43.08, 71.52]	[14.85, 43.73]	[9.28, 43.28]	[35.87, 69.21]
Subscale 3: Fear of loss of time & opportunity	41.69 ± 8.63	29.67 ± 4.16	26.39 ± 7.36	46.18 ± 5.59
	[24.38, 59.00]	[21.33, 38.01]	[11.62, 41.16]	[33.84, 58.53]
Subscale 4: Fear of an uncertain future	54.39 ± 7.22	26.56 ± 6.66	28.13 ± 7.90	55.20 ± 7.99
	[39.36, 69.41]	[12.80, 40.31]	[12.17-, 4.09]	[37.78, 72.62]
Subscale 5: Fear of suffering & pain	66.12 ± 5.99	42.10 ± 6.28	40.83 ± 7.62	68.21 ± 6.56
	[53.50, 78.74]	[29.22, 54.99]	[25.31, 56.35]	[53.81, 82.62]
Subscale 6: Fear of sudden death	50.67 ± 6.92	24.48 ± 7.23	20.10 ± 8.32	44.76 ± 8.32
	[35.79, 65.55]	[9.62, 39.33]	[3.28, 36.93]	[26.49–63.02,

Abbreviation: DADDS, Death and Dying Distress Scale.

All adjusted change calculated from individual baseline to account for participant baseline variability.

^ Within <30 days of deceased date.

**Figure 1.**
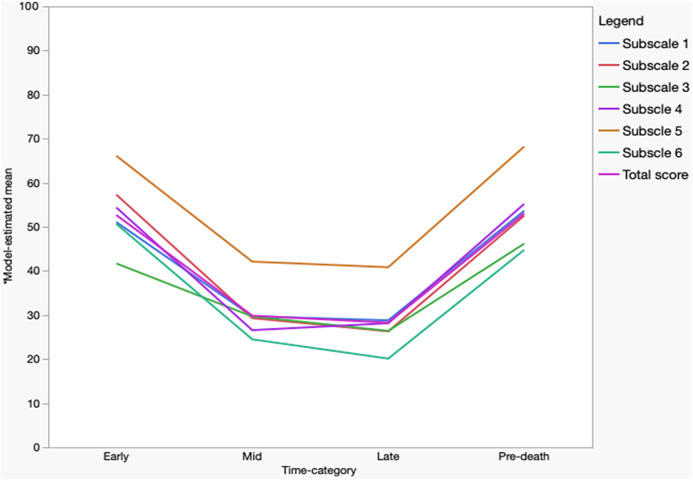
Visual representation of model derived mean DADDS scores over time.

All six subscales demonstrated statistically significant time effects (p < 0.05), each following a similar temporal trend (see Fig. 1). *Fear of suffering and pain* was consistently the highest-rated subscale (early: 66%, mid: 42%, late: 41%, near-death: 68%). The lowest subscale varied by stage: *fear of loss of time and opportunity* in early (42%) and *fear of sudden death* in mid (24%), late (20%), and near-death (45%) stages. The largest decline from early to mid-stage was observed in *fear of the dying process* (β = −28.01, p = 0.007), and the least in *fear of loss of time and opportunity* (β = −12.02, p = 0.302, NS). The largest increase from late to near-death occurred in *fear of suffering and pain* (β = 27.38, p = 0.006). No subscale showed statistically significant change between mid and late stages. See Tables 3 and 4 for all mixed-effects model coefficients and the model-derived estimates, respectively.

A Tukey-adjusted post-hoc comparison indicated that DADDS total scores were significantly higher in the near-death stage than the mid stage (Δ = 23.20, 95% CI [2.50, 43.90], p = .023. The comparison between the near-death and early stages was not statistically significant (Δ = 0.36, 95% CI [–19.72, 20.44], p = 1.000).

### Distress and survival

A Weibull parametric survival model, controlling for age, gender, diagnosis, phase of care, and duration of palliative care prior to study enrolment, showed that each one-point increase in average distress score change during the study period was associated with a time ratio of 0.9300 (β = −0.0703; 95% CI: −0.110 to −0.030; p < 0.001), indicating a 6.97% reduction in survival time measured from study start. Based on the modelled expected survival of 174 days and the observed mean survival of 353 days (SD 292), this equates to approximately 12 and 25 fewer days of survival, respectively, per one-point increase in distress. These findings suggest that independent of key demographic and clinical factors, greater distress during the study was significantly associated with shorter survival from study enrolment.

## Discussion

This study contributes valuable insights into psychosocial distress patterns among rural palliative care patients, a population underrepresented in current literature. A U-shaped distress trajectory was identified, with peaks early in care and near death, highlighting the complex, non-linear emotional challenges during these transitions. Although raw mean scores suggested a late-stage maximum, this was likely due to changes in sample composition rather than true within-person change; modelled analyses confirmed a genuine U-shaped pattern. These findings suggest that distress fluctuates with disease progression and stabilizes during structured, supportive phases, aligning with prior research linking distress to symptom burden and uncertainty (Rodin et al. 2009; Walbaum et al. 2024). All six DADDS domains showed significant temporal variation, reinforcing distress’s multidimensional nature and the need for phase-specific interventions. The study also found elevated distress significantly associated with shorter survival, supporting Smith et al.’s (Smith et al. 2021) proposal that distress may be a clinically relevant prognostic indicator requiring early identification and management. While patterns broadly mirrored those demonstrated in urban cohorts, rural-specific challenges, such as limited local resources and inconsistent access to psychosocial support (Parliament of NSW 2022), may intensify distress and emphasize the need for tailored, context-sensitive support during peak periods.

Subscale trajectories broadly followed the overall U-shape but revealed important distinctions in how distress escalated, resolved, or persisted across illness phases. Fear of suffering and pain consistently emerged as the most prominent distress domain throughout palliative care, highlighting the ongoing impact of both actual and anticipated suffering, especially in rural contexts where access to specialist care and timely symptom relief is often limited (Parliament of NSW 2022). Conversely, fear of sudden death remained the least endorsed subscale across most stages, supporting the notion that familiarity with disease progression and ongoing care engagement may reduce fear of the unknown and improve emotional preparedness (Walbaum et al. 2024). Fear of lost time and missed opportunities remained relatively stable over time, consistent with prior findings identifying it as a central concern and suggesting it may be a persistent issue inadequately addressed by standard care (Walbaum et al. 2024). These findings reinforce the importance of palliative care approaches that extend beyond symptom control to embrace meaning-oriented, person-centered care.

The early stage of palliative care featured a pronounced peak in distress, primarily driven by fear of suffering and pain. This likely reflects the acute symptom burden at care initiation combined with uncertainty about disease trajectory, both well-established contributors to early distress (Walbaum et al. 2024). Among rural patients, these fears may be amplified by logistical challenges such as travel demands and limited local expertise (Parliament of NSW 2022). Early psychosocial care should therefore prioritize anticipatory guidance, emotional support, and structured advance care planning to help patients manage uncertainty and regain a sense of control.

Similarly, the final 30 days before death saw a significant resurgence of distress across all subscales, most notably fear of suffering and pain, which increased by 27.4 percentage points to reach 68.2%. This aligns with literature attributing heightened awareness of mortality and worsening symptoms as key drivers in end-of-life distress (Rodin et al. 2009; Walbaum et al. 2024). In contrast, *fear of loss of time and opportunity* showed the least increase during this time, suggesting that although this domain continues to rise near death, it may become less central as more immediate concerns take precedence. These observations underline the need for phase-specific psychosocial interventions in the final weeks of life, such as anticipatory symptom management, existential counselling, and emotionally attuned support, to mitigate distress associated with a final transition towards end-of-life.

Between these peaks, distress declined significantly during the mid-stage, primarily driven by reductions in fear of the dying process. This supports the idea that ongoing palliative care engagement fosters acceptance, demystifies death, and alleviates anxiety. As Hildenbrand et al. observed, psychological adaptation may occur through structured care, fostering a sense of control and acceptance (Hildenbrand et al. 2022). However, fear of loss of time and opportunity showed the smallest, statistically non-significant decline, suggesting that such existential concerns are less responsive to routine care. This gap underscores the value of meaning-centered interventions, such as legacy work, life review, or values-based goal setting, with similar dignity therapy shown to reduce distress and enhance quality of life in patients nearing end-of-life (Seiler et al. 2024). Meanwhile late-stage plateau in distress with negligible further decline, may reflect either a psychological ceiling effect where further improvements become difficult or a sustained benefit from earlier support, consistent with research proposing psychological adaptation from structured care (Hildenbrand et al. 2022). Nevertheless, fear of suffering and pain again remained the dominant concern, underscoring the need for consistent symptom management and ongoing psychosocial support throughout care, ideally mitigating before it intensifies in the final 30 days. Providing patients with regular opportunities to express fears through supportive communication may help address persistent distress, as such approaches have been demonstrated to improve emotional well-being in advanced illness (Lo et al. 2014).

Importantly, the survival analysis findings indicated that higher psychosocial distress during the study period corresponded with significantly fewer survival days. Although causality cannot be confirmed, these findings suggest distress as a meaningful clinical indicator with potential prognostic value. This aligns with a meta-analysis by Smith et al. (2021), which reported modest survival benefits from psychosocial interventions but noted considerable variability across patient populations and care contexts. While disease-specific research is needed to clarify who benefits most and under what circumstances, possible mechanisms may include psychoneuroimmunological pathways, whereby chronic psychological stress impairs immune function and accelerates disease progression, or by indirect effects like reduced care engagement, lower treatment adherence, or increased symptom burden and unmet needs (Kiecolt-Glaser and Glaser 1999; Khalid et al. 2023). These results reinforce the importance of holistic palliative care approaches that address both emotional and physical wellbeing to optimize patient outcomes (Smith et al. 2021).

### Clinical implications

This study identifies two critical windows for intervention: the early stage of palliative care and the final 30 days of life. Early interventions should focus on mitigating fears related to uncertainty, loss of control, and the progression of disease through timely information provision, advance care planning, and psychological support. Nearing death, care should prioritize managing existential fears, actual and anticipatory physical symptoms, and autonomy loss. Given the prominence of fears regarding suffering, psychoeducational strategies, such as patient involvement in care decisions, anticipatory guidance and open, compassionate discussions about the disease and dying process are likely beneficial, especially in rural settings where psychological support resources may be scarce. In addition, the potential role of psychosocial distress as a clinical indicator underscores the importance of implementing routine distress screening and proactive psychosocial care throughout the palliative trajectory. Scalable rural approaches might include telehealth counselling, community support programs, and capacity building among local healthcare providers to ensure contextually appropriate care.

### Limitations and future research

Limitations include the small sample size and single-site design limiting generalizability. Variability in enrolment timing and irregular observation schedules, while statistically managed, reflect real-world challenges of longitudinal palliative care research. Absence of longitudinally tracked clinical covariates (e.g., functional status) and lack of qualitative data limit deeper contextual understanding.

Future research should involve larger, multisite rural cohorts with longitudinal tracking of clinical indicators and standardized assessment intervals, ideally beginning earlier in the palliative trajectory. Qualitative studies could complement findings by exploring rural patients lived experiences and meanings of death-related distress. Research to elucidate the pathways that may be involved in psychosocial distress and shorter predicted survival time would be of benefit, as would interventional trials targeting early and end-of-life stages to refine evidence-based rural palliative care.

## Conclusion

This study demonstrates the non-linear, U-shaped trajectory of psychosocial distress in rural palliative care and identifies early and pre-death stages as periods of heightened vulnerability. The link between distress and reduced survival underscores the clinical importance of addressing psychosocial needs comprehensively. While disparities in rural palliative care delivery are well recognized, these findings offer novel insights into the burden of psychosocial distress in these populations and highlight the necessity of developing contextually appropriate, evidence-informed interventions tailored to rural populations for equitable, patient-centered end-of-life care.
